# Different pain phenotypes are associated with anti-Caspr2 autoantibodies

**DOI:** 10.1007/s00415-024-12224-4

**Published:** 2024-02-22

**Authors:** Patrik Greguletz, Maria Plötz, Carolin Baade-Büttner, Christian G. Bien, Katharina Eisenhut, Christian Geis, Robert Handreka, Jaqueline Klausewitz, Peter Körtvelyessy, Stjepana Kovac, Andrea Kraft, Jan Lewerenz, Michael Malter, Michael Nagel, Felix von Podewils, Harald Prüß, Anna Rada, Johanna Rau, Sebastian Rauer, Rosa Rößling, Thomas Seifert-Held, Kai Siebenbrodt, Kurt-Wolfram Sühs, Simone C. Tauber, Franziska Thaler, Judith Wagner, Jonathan Wickel, Frank Leypoldt, Heike L. Rittner, Claudia Sommer, Carmen Villmann, Kathrin Doppler, the GENERATE study group, Michael Adelmann, Luise Appeltshauser, Ilya Ayzenberg, Andreas van Baalen, Sebastian Baatz, Oliver Bähr, Bettina Balint, Sebastian Bauer, Annette Baumgartner, Stefanie Becker, Sonka Benesch, Robert Berger, Birgit Berger, Martin Berghoff, Sascha Berning, Sarah Bernsen, Achim Berthele, Christian Bien, Corinna Bien, Andreas Binder, Stefan Bittner, Daniel Bittner, Franz Blaes, Astrid Blaschek, Amelie Bohn, Sergio Castro-Gomez, Justina Dargvainiene, Timo Deba, Julia Maren Decker, Andre Dik, Juliane Dominik, Mona Dreesmann, Friedrich Ebinger, Lena Edelhoff, Laura Ehrhardt, Sven Ehrlich, Alexander Emmer, Dominique Endres, Marina Entscheva, Daniela Esser, Thorleif Etgen, Jürgen Hartmut Faiss, Kim Kristin Falk, Walid Fazeli, Alexander Finke, Carsten Finke, Dirk Fitzner, Marina Flotats-Bastardas, Mathias Fousse, Tobias Freilinger, Paul Friedemann, Manuel Friese, Marco Gallus, Marcel Gebhard, Anna Gorsler, Armin Grau, Oliver Grauer, Britta Greshake, Catharina Groß, Thomas Grüter, Aiden Haghikia, Niels Hansen, Jens Harmel, Antonia Harms, Yetzenia Dubraska Haro Alizo, Martin Häusler, Joachim Havla, Chung Ha-Yeun, Wolfgang Heide, Valentin Held, Kerstin Hellwig, Philip Hillebrand, Frank Hoffmann, Christian Hofmann, Ulrich Hofstadt-van Oy, Peter Huppke, Hagen Huttner, Fatme Seval Ismail, Martina Jansen, Mareike Jansen, Aleksandra Juranek, Michael Karenfort, Max Kaufmann, Christoph Kellinghaus, Constanze Kerin (geb. Mönig), Susanne Knake, Ellen Knierim, Peter Körtvélyessy, Markus Krämer, Verena Kraus, Christos Krogias, Gregor Kuhlenbäumer, Tanja Kümpfel, Christoph Lehrich, Andeas Linsa, Jan Lünemann, Marie Madlener, Niels Margraf, Carlos Martinez Quesada, Monika Meister, Nico Melzer, Kristin Stefanie Melzer, Til Menge, Sven Meuth, Gerd Meyer zu Hörste, Fabian Möller, Marie-Luise Mono, Sigrid Mues, Jost Obrocki, Loana Penner, Lena Kristina Pfeffer, Thomas Pfefferkorn, Steffen Pfeuffer, Alexandra Philipsen, Johannes Piepgras, Felix von Poderwils, Mosche Pompsch, Josef Priller, Anne-Katrin Pröbstel, Daniel Rapp, Dominica Ratuszny, Johanna Maria Helena Rau, Saskia Jania Räuber, Robert Rehmann, Ina Reichen, Gernot Reimann, Raphael Reinecke, Nele Retzlaff, Marius Ringelstein, Henrik Rohner, Felix Rosenow, Kevin Rostasy, Theodor Rüber, Stephan Rüegg, Yannic Saathoff, Jens Schaumberg, Ruth Schilling, Mareike Schimmel, Jens Schmidt, Ina-Isabelle Schmütz, Hauke Schneider, Patrick Schramm, Stephan Schreiber, Gesa Schreyer, Ina Schröder, Simon Schuster, Günter Seidel, Frank Seifert, Makbule Senel, Olga Simova, Juliane Spiegler, Oliver Stammel, Andeas Steinbrecher, Henning Stolze, Muriel Stoppe, Karin van`s Gravesande Storm, Christine Strippel, Dietrich Sturm, Klarissa Hanja Stürner, Steffen Syrbe, Pawel Tacik, Simone Tauber, Florian Then Bergh, Anja Tietz, Corinna Trebst, George Trendelenburg, Regina Trollmann, Thanos Tsaktanis, Hayrettin Tumani, Methap Türedi, Christian Urbanek, Niklas Vogel, Max Vogtmann, Matthias von Mering, Jan Wagner, Klaus-Peter Wandinger, Robert Weissert, Brigitte Wildemann, Karsten Witt, Kartharina Wurdack, Lara Zieger

**Affiliations:** 1 Department of Neurology, University Hospital Würzburg, Josef-Schneider-Str. 11, 97080 Würzburg, Germany; 2 Institute of Clinical Neurobiology, University Hospital Würzburg, Würzburg, Germany; 3 Section Translational Neuroimmunology, Department for Neurology, Jena University Hospital, Jena, Germany; 4 Department of Epileptology (Krankenhaus Mara), Medical School, Bielefeld University, Campus Bielefeld-Bethel, Bielefeld, Germany; 5 Laboratory Krone, Bad Salzuflen, Germany; 6 Institute of Clinical Neuroimmunology, University Hospital, Ludwig-Maximilians-Universität Munich, Munich, Germany; 7 Biomedical Center (BMC), Medical Faculty, Ludwig-Maximilians-Universität Munich, Martinsried, Germany; 8 Munich Cluster for Systems Neurology (SyNergy), Munich, Germany; 9 Carl-Thiem-Klinikum, Cottbus, Germany; 10 Department of Neurology, St. Josef-Hospital, Ruhr-University Bochum, Bochum, Germany; 11 Department of Neurology, University Hospital Magdeburg, Magdeburg, Germany; 12 Department of Neurology with Institute of Translational Neurology, University Hospital Münster, Münster, Germany; 13 Department of Neurology, Martha-Maria Hospital Halle, Halle, Germany; 14 Department of Neurology, University of Ulm, Ulm, Germany; 15 Department of Neurology, Faculty of Medicine and University Hospital Cologne, University of Cologne, Cologne, Germany; 16 Department of Neurology, Klinikum Osnabrück, Osnabrück, Germany; 17 Department of Neurology, University Hospital Greifswald, Greifswald, Germany; 18 Department of Neurology and Experimental Neurology, Charité Berlin, and German Center for Neurodegenerative Diseases (DZNE),, Berlin, Germany; 19 Department of Neurology, University of Freiburg, Freiburg im Breisgau, Germany; 20 Department of Neurology, Medical University of Graz, Graz, Austria; 21 Department of Neurology, Hospital Murtal, Knittelfeld, Austria; 22 Department of Neurology, University Hospital Frankfurt, Frankfurt, Germany; 23 Department of Neurology, Hannover Medical School, Hannover, Germany; 24 Department of Neurology, RWTH University Hospital Aachen, Aachen, Germany; 25 Department of Neurology, Kepler University Hospital Linz, Linz, Austria; 26 Department of Neurology, Evangelisches Klinikum Gelsenkirchen, Academic Hospital University Essen-Duisburg, Gelsenkirchen, Germany; 27 Department of Neurology, Christian-Albrechts-University Kiel, Kiel, Germany; 28 Department of Anesthesiology, Intensive Care, Emergency Medicine and Pain Medicine, Centre for Interdisciplinary Pain Medicine, University Hospital Würzburg, Würzburg, Germany

**Keywords:** Caspr2, Pain, Autoantibody, IgG subclass, IgG4

## Abstract

Autoantibodies against contactin-associated protein 2 (Caspr2) not only induce limbic autoimmune encephalitis but are also associated with pain conditions. Here, we analyzed clinical data on pain in a large cohort of patients included into the German Network for Research in Autoimmune Encephalitis. Out of 102 patients in our cohort, pain was a frequent symptom (36% of all patients), often severe (63.6% of the patients with pain) and/or even the major symptom (55.6% of the patients with pain). Pain phenotypes differed between patients. Cluster analysis revealed two major phenotypes including mostly distal-symmetric burning pain and widespread pain with myalgia and cramps. Almost all patients had IgG4 autoantibodies and some additional IgG1, 2, and/or 3 autoantibodies, but IgG subclasses, titers, and presence or absence of intrathecal synthesis were not associated with the occurrence of pain. However, certain pre-existing risk factors for chronic pain like diabetes mellitus, peripheral neuropathy, or preexisting chronic back pain tended to occur more frequently in patients with anti-Caspr2 autoantibodies and pain. Our data show that pain is a relevant symptom in patients with anti-Caspr2 autoantibodies and support the idea of decreased algesic thresholds leading to pain. Testing for anti-Caspr2 autoantibodies needs to be considered in patients with various pain phenotypes.

**Supplementary Information:**

The online version contains supplementary material available at 10.1007/s00415-024-12224-4.

## Introduction

Autoantibodies against contactin-associated protein 2 (Caspr2) are associated with a variety of clinical phenotypes including limbic encephalitis, neuromyotonia, cerebellar dysfunction, dysautonomia, insomnia, movement disorders, and neuropathic pain [1–4]. The autoantibodies mostly belong to the IgG4 subclass that does neither induce complement deposition, activation of inflammatory cells, nor internalization of surface proteins, but additional IgG1 autoantibodies have also been described [3]. Caspr2 is part of the voltage-gated potassium channel complex that modulates neuronal excitability [5]. Neuropathic pain is supposed to be induced by binding of anti-Caspr2 autoantibodies to dorsal root ganglia (DRG) neurons, resulting in hyper-excitability of nociceptive neurons [6]. However, neuropathic pain is only reported in about 30–60% of all patients with anti-Caspr2 autoantibodies, and the phenotype of pain varies [2, 4, 7–9]. Individual differences either of the patients or at the autoantibody level may account for differences in the induction of neuropathic pain.

In the present study, a large multicenter cohort of patients with anti-Caspr2 autoantibodies was screened for neuropathic pain. The clinical phenotypes of patients with and without neuropathic pain as well as IgG subclass distribution and autoantibody titers were systematically assessed and compared.

## Methods

### Study cohort

The registry of the German Network for Research on Autoimmune Encephalitis (GENERATE), a multicenter database for patients with autoimmune encephalitis in Germany, Austria, and Switzerland (generate-net.de) was searched for patients who had been positively tested for anti-Caspr2 autoantibodies at the participating centers between 2011 and 2023, resulting in 115 datasets of patients. All datasets were manually checked for plausibility. Five patients were finally excluded due to lacking information on pain symptoms, eight were excluded because of very low anti-Caspr2 titers (1:10 or less). Autoantibody testing was performed at different laboratories, all using cell-based assays. The antibody index was calculated according to Reiber et al., a cut-off value of > 4 was applied for intrathecal synthesis [10].

All patients gave written informed consent to be enrolled in the GENERATE registry and the registry was approved by the Ethic committees of all participating centers.

All recruiting centers were contacted and asked for information on pain in patients treated at their hospital. Only patients whose records contained information on pain were finally included into the study, resulting in a study cohort of 102 patients. All centers were asked to retrospectively extract the following information on pain from the patient records: localization, quality, intensity, temporal course, relieving/deteriorating factors, major symptom (yes/no), and response to immunosuppressive treatment (mostly glucocorticoids and/or rituximab). In most hospitals, pain intensity was scaled from 0 to 10 on the numeric rating scale. From some patients, no numeric pain rating was available, and pain was only categorized as “mild”, “moderate”, or “severe” in the patient records. We therefore decided to use these three categories and the values from the numeric rating scale were categorized as mild (1–3), moderate (4–6), or severe (7–10) accordingly. Additionally, information regarding the following conditions was requested: neuromyotonia, peripheral neuropathy, and diabetes mellitus.

### Detection of anti-Caspr2 autoantibodies and IgG subclass analysis

Sera for the analysis of IgG subclasses were available from 48 of the included patients. First, the presence of autoantibodies against Caspr2 in the sera was validated via cell-based assays (CBA) using human embryonic kidney 293 (HEK293, (CRL-1573; ATCC – Global Bioresource Center, Manassas, VA, USA) cells. After one day of culturing at 37 °C and 5% CO_2_, the cells were transfected with the Caspr2 plasmid (kindly provided by J. Dalmau [11]) using calcium phosphate precipitation as described before [12]. After two days, the transfected cells were incubated with patient serum (1:250, one serum that was negative at 1:250 was repeated with a dilution of 1:100) and a commercial anti-Caspr2 antibody from sheep (1:250, R&D Systems by Bio-Techne, AF5145) for one hour. Then, the cells were fixed with 4% PFA and 4% sucrose in PBS (pH 7.4) for 20 min on ice. Blocking with 5% horse serum in PBS (pH 7.4) at room temperature was performed for 30 min. Afterward, the cells were incubated with donkey anti-sheep Alexa Fluor 488 (Jackson ImmunoResearch, 713–545-147) and goat anti-human Cy3 (Jackson ImmunoResearch, 109–165-003) secondary antibodies diluted 1:500 in PBS (pH 7.4) for an hour at room temperature. The coverslips were incubated with DAPI (4’, 6-Diamidino-2-phenylindol, 1:5000) for 5 min and then mounted with Mowiol.

For the IgG subclass determination, the CBA described above was conducted using IgG subclass-specific secondary antibodies. Initially, the cells were exposed to patient serum and serum of healthy controls with a dilution of 1:250. Subclass determination for sera with low titers was exhibited with a 1:50 or the lowest 1:25 dilution of the patient sera. FITC- or Alexa Fluor 488-conjugated commercial secondary antibodies at a dilution of 1:100 were applied (anti-human IgG1: Abcam, ab99772; anti-human IgG2: Southern Biotech, 9070–30; anti-human IgG3: Sigma Aldrich, F4641; anti-human IgG4: Abcam, ab99815). As controls, coverslips with untransfected cells were incubated with serum and goat anti-human Cy3 secondary antibody or stained with commercial anti-Caspr2 primary antibody from sheep and donkey anti-sheep Alexa Fluor 488 as the secondary antibody.

### Statistical analysis

All statistical tests were performed using R version 3.4.1. Polytomous Latent Class analysis for clustering was performed using R package poLCA (version 1.6.0).

The motivation for Polytomous Latent Class analysis was that this method allows to identify and estimate if the underlying distribution is a mixture or not. This was achieved using the Akaike Information Criterion (AIC) for model order selection. If the model with the best (minimal) AIC has more than one component, this characterizes clusters of observations coming from the same distribution.

For each number of clusters, the minimum AIC of 25 runs was calculated. Optimal number of components was determined by the minimum AIC.

Cluster analysis included the following dichotomous variables: distal > proximal pain, back pain, myalgia/arthralgia, high intensity, increase with exercise, burning pain, muscle soreness, cramps, tearing pain, dysesthesia, dull pain, temperature-dependent pain, pain as major symptom, no response to treatment. For comparison of metric data, t tests were performed, for categorical data, chi-square test was used, and a significance level of < 0.05 was applied in all tests.

## Results

### Patient cohort

**Fig. 1:**
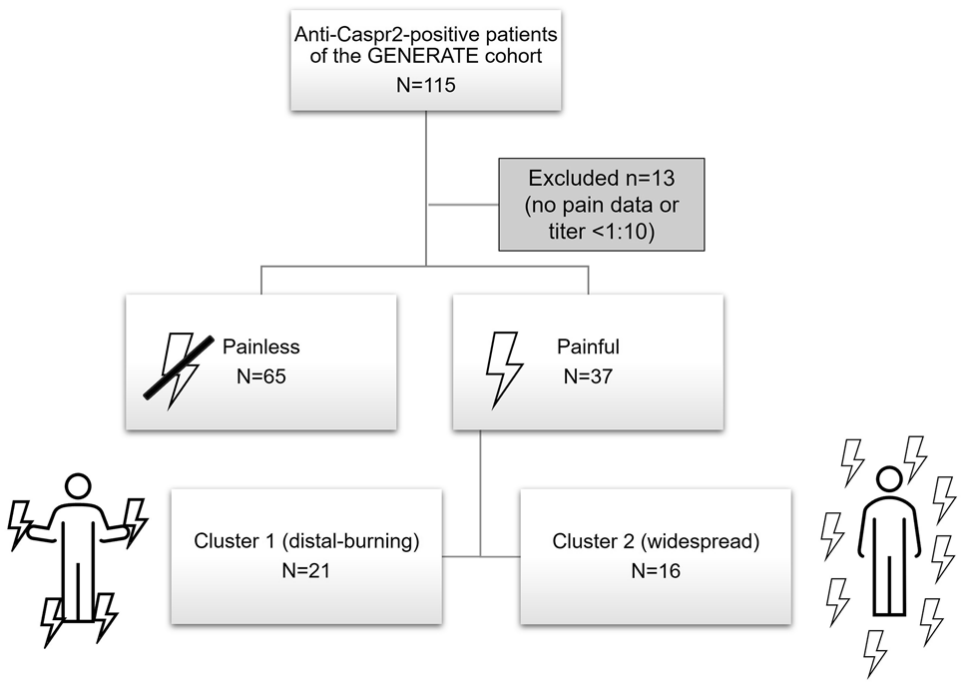
Flow chart illustrating the composition of the study cohort. The whole cohort can be divided into painless and painful phenotypes, patients with painful disease were further subdivided into two clusters

Of the 102 anti-Caspr2-positive patients who were finally included, 37 reported chronic pain, 65 did not report any pain (Fig. 1). Only pain that occurred for the first time at the onset of anti-Caspr2-associated disease or clearly exacerbated in temporal relation with the disease was considered, no other chronic pain states. Median age, sex and autoantibody titers are summarized in Table 1 (left two columns) and did not differ between both groups.

**Table 1:** Overview on demographic data, IgG subclasses, CSF findings and concomitant diseases in anti-Caspr2-positive patients with pain and without pain

	No pain (*n* = 65)	Pain (*n* = 37)	Pain cluster 1 (*n* = 21)	Pain cluster 2 (*n* = 16)
Mean age (SD)	62.1 (± 14.4)	60.8 (± 14.3)	60.4 (± 14.7)	61.3 (± 14.7)
Sex (male)	58 (89%)	32 (86.5%)	19/21 (90.5%)	13/16 (81.2%)
IgG subclass				
IgG4 only	6/21 (28.6%)	4/19 (21.1%)	3/14 (21.4%)	1/5 (20%)
IgG4 + IgG1-3 or IgG1-3 only	15/21 (71.4%)	15/19 (78.9%)	11/14 (78.6%)	4/5 (80%)
Serum/CSF anti-Caspr2- positive	64/65 (98.5%) // 38/49 (77.5%)	36/37 (97.3%) // 21/30 (70%)	21/21 (100%) // 13/18 (72.2%)	15/16 (93.8%) // 8/12 (66.7%)
Median titer serum/CSF (range)	1:1000 (1:50–1:32,000)/1:320 (1:10–1:3200)	1:1000 (1:32–1:10,000)/1:100 (1:1–1:10,000)	1:1000 (1:32–1:10,000)/1:100 (1:1–1:10,000)	1:550 (1:32–1:3200)/1:100 (1:100–1:320)
Intrathecal anti-Caspr2 synthesis	23/43 (53.5%)	16/30 (53.3%)	11/18 (61.1%)	5/12 (41.7%)
Pleocytosis	27/54 (50%)	10/35 (28.6%)	8/20 (40%)	2/15 (13.3%)
Diabetes m.				
Prevalence in the general population 22% (male, 60–65 years) [18]	10/65 (15.4%)	6/37 (16.2%)	5/21 (23.8%)	1/16 (6.2%)
PNP				
Prevalence in the general population 14.6% (male, mean 70 years) [19]	16/62 (29%)*	20/36 (55.5%)*	14/20 (70%)	6/16 (37.5%)
Preexisting chronic back pain/spinal surgery				
Prevalence in the general population 16.7% (male, 60–69 years) [20]	8/65 (12.3%)	9/37 (24.3%)	5/21 (23.8%)	4/16 (25%)
Neuromyotonia	3/62 (4.8%)*	12/37 (32.4%)*	6/21 (28.6%)	6/16 (37.5%)

*SD* standard deviation, *CSF* cerebrospinal fluid, *PNP* peripheral neuropathy

*p < 0.05 (Chi square test)

78 of the patients (51 without pain, 27 with pain) had symptoms of limbic encephalitis and fulfilled the clinical diagnostic criteria of possible autoimmune encephalitis according to Graus et al. [13] (and of course definite autoimmune encephalitis when considering the positive anti-Caspr2 findings). The others did not show any cognitive, mental, or psychiatric symptoms but other symptoms indicating anti-Caspr2-associated disease like cerebellar dysfunction, seizures, neuromyotonia, or autonomic symptoms.

### Autoantibody titers and distribution

Serum and cerebrospinal fluid (CSF) titers of diagnostic anti-Caspr2 testing were retrieved from the GENERATE database or the patients records and were available in 48 patients without pain and 37 patients with pain, all tested by assays from Euroimmun (Lübeck, Germany). The remaining 17 patients were clearly positive for anti-Caspr2 with other tests and/or the initial titer could not be reported by the recruiting center. Of the patients without pain, 37/48 (77.1%) were anti-Caspr2-positive in the serum and CSF, 10/48 (20.8%) were only positive in the serum and 1/48 (2.1%) only in the CSF. Of the anti-Caspr2-positive patients with pain, 21/37 (56.8%) were positive in the serum and CSF, 9/37 (24.3%) were only positive in the serum and none of the patients only in the CSF. In seven patients, no information of CSF titer was available. Titers did not differ between patients with and without pain (Table 1, left two columns). The number of patients with CSF pleocytosis was also similar in patients with and without pain (even if there was a slight trend toward more patients with pleocytosis in the cohort without pain (Table 1)). The median time between symptom onset and lumbar puncture was 4 months in both groups (range 0–197 months (without pain) and 0–106 (with pain)) and did not differ between groups. There was no correlation between pleocytosis and the time between symptom onset and CSF analysis. Intrathecal autoantibody production (based on the autoantibody index) could be assessed in 43 patients without pain and 30 patients with pain. Intrathecal production could be found in 23/43 (53.5%) of the patients without pain and 16/30 (53.3%) with pain. Thus, neither the autoantibody titers nor the distribution of autoantibodies between compartments (CSF vs. serum) could be clearly attributed to painful or painless manifestations.

### IgG subclasses in patients with/without pain

**Fig. 2:**
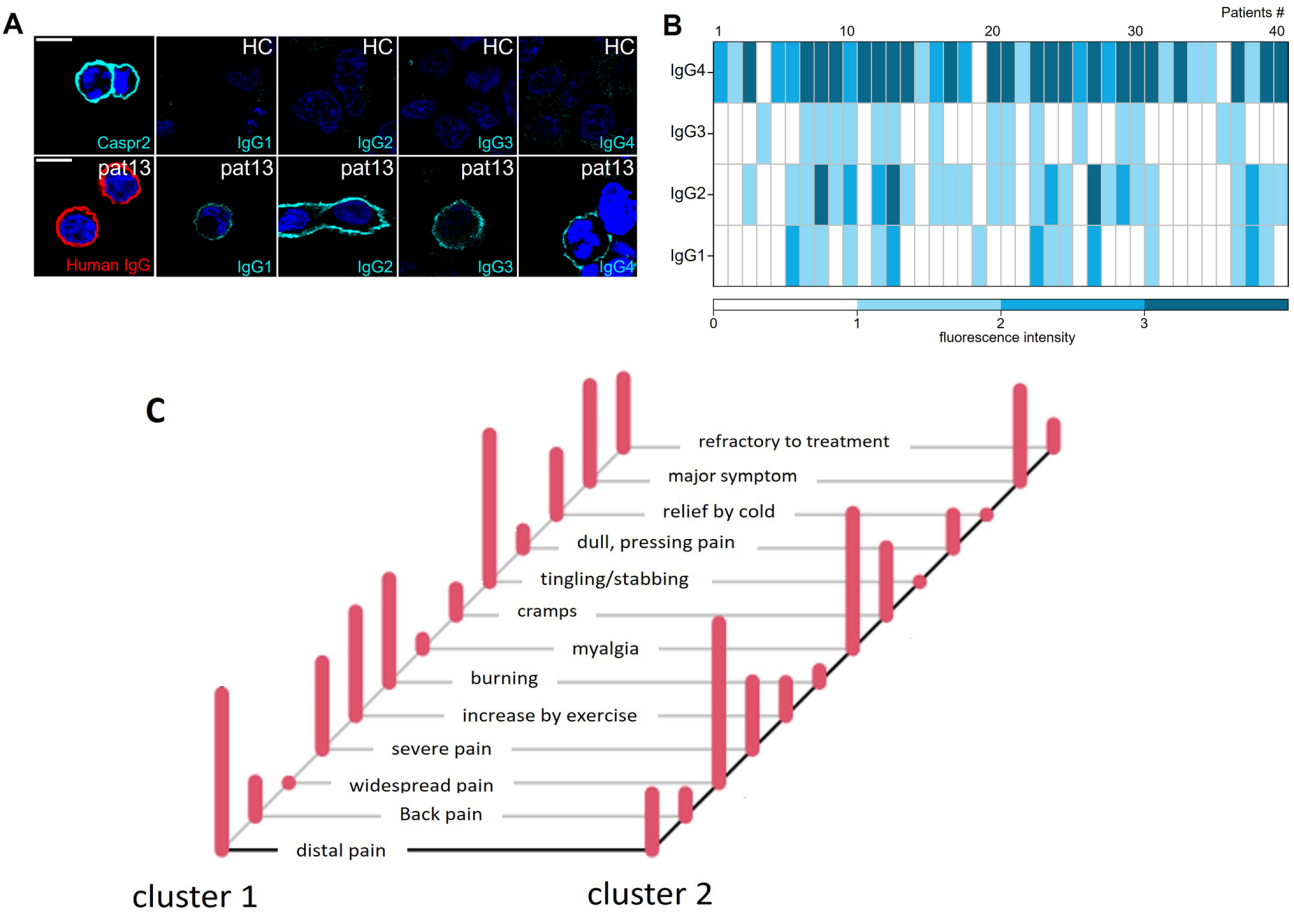
Patient anti-Caspr2 autoantibodies show distinct IgG subclass pattern independent of the pain phenotype. HEK293 cells were transfected with human Caspr2. **A** Caspr2 was stained with a commercial antibody (1:250; cyan; upper left image). Secondary FITC-coupled IgG subclass antibodies (IgG1-4) were tested with healthy control (HC, 1:250) serum instead of patient (pat) serum as negative controls (upper lane, right images). Representative images following incubation with human patient serum 13 (1:250) detected by secondary antibodies against total human IgG (red; lower left image). Serum of patient 13 (1:50 for subclass determination) was positive for all four IgG subclasses but with different intensities (IgG3 < IgG1 < IgG2 = IgG4; cyan, lower right images). DAPI marks the nuclei. Scale bar refers to 10 µm. **B** Intensity plot of IgG subclass determination from all patients (40 total) investigated. Intensities were classified from 0–1-2–3 (white—light blue—blue—dark blue; no staining—very weak but visible staining—intense staining—very intense staining). Note, almost all sera contained Caspr2 autoantibodies of subclass IgG4. In (**C**), the probability of different variables of cluster analysis in cluster 1 and 2 is depicted. Patients of cluster 1 suffer from mostly distal burning and tingling pain that is increased by exercise and cold whereas cluster 2 is characterized by widespread pain, myalgia and cramps. *pat* patient, *HC* healthy control

IgG subclasses of anti-Caspr2 could be determined in sera of 40 patients, 21 patients without pain and 19 patients with pain (Fig. 2A, B, Table 1 left two columns). In sera of eight patients (all with low titers of anti-Caspr2), no subclass was detectable, i.e., all CBA with subclass-specific secondary antibodies were negative, most probably because of the low titer.

IgG4 autoantibodies were detectable in all sera except for two patients (both without pain) where only IgG3 anti-Caspr2 autoantibodies could be found and one serum of a patient with pain where only IgG1 was detectable. In 6/21 (28.6%) sera of patients without pain, IgG4 was the only autoantibody subclass, in 13/21 (61.9%), IgG4, and autoantibodies of other subclasses (IgG1, IgG2 and/or IgG3) were detectable. Of the patients with pain, 4/19 (21.1%) had IgG4 autoantibodies only, in 14/19 (73.7%) additional other subclasses were detectable.

### Two major pain phenotypes associated with anti-Caspr2 autoantibodies

In the patients with anti-Caspr2 autoantibodies and pain, information on the severity of pain was available in 22 patients and was reported as “severe” by most of them (14/22), as “moderate” by 6/22 and as mild by only two patients. Pain was the major symptom in 20/37 patients who experienced pain and the only symptom in two patients. When evaluating the descriptions of the pain phenotypes of the anti-Caspr2-positive patients, different phenotypes became apparent: Some patients suffered from distal-symmetric burning pain and/or allodynia, corresponding to the pain phenotype typically found in patients with small fiber neuropathy. Other patients experienced severe back pain radiating to the legs, resembling radiculitis and other patients reported chronic widespread pain including myalgia and arthralgia, in some cases accompanied by muscle cramps.

To better differentiate pain phenotypes, cluster analysis including localization, severity, provoking factors, and clinical description of pain was performed using model order selection. Four clusters were proposed, but analysis was adapted to two clusters due to plausibility and because otherwise the groups would have been too small for further comparison. In cluster 1 (*n* = 21), pain was located at the distal legs and feet and/or in the back, was often burning or tingling, sometimes relieved by cooling (or aggravated by heat) and was often difficult to treat, thus resembling neuropathic pain experienced in small fiber neuropathy (see Table 2, Fig. 2C). Patients of cluster 2 (*n* = 16) reported more widespread pain, often myalgia and/or muscle cramps that often responded to treatment (Table 2). Age, sex, autoantibody titers, CSF findings, and IgG subclasses were not different between the clusters (Table 1, right two columns).

**Table 2:** Pain characteristics of anti-Caspr2-positive patients

	Pain (all) (*n* = 37)	Pain cluster 1 (*n* = 21)	Pain cluster 2 *(n* = 16)
Intensity (mild/moderate/severe)	2/6/14 of 22 (9.1%/27.3%/ 63.6%)	2/3/8 of 13 (15.4%/23.1%/ 61.5%)	0/3/6 of 9 (0%/33.3%/66.7%)
Localized (feet and/or legs /back)	27/37 (73%)	21/21 (100%)	6/16 (37.5%)
Widespread	10/37 (27%)	0	10/16 (62.5%)
Burning	14/36 (38.9%)	12/21 (57.1%)	1/16 (6.3%)
Tingling or stabbing	11/36 (30.6%)	17/21 (81%)	0
Myalgia/arthralgia	8/36 (22.2%)	1/21 (4.8%)	12/16 (75%)
Cramps	7/36 (19.4%)	3/21 (14.3%)	6/16 (37.5%)
Relief by cold, increase by heat	7/37 (18.9%)	7/21 (33.3%)	0
Increased by exercise	13/37 (35.1%)	12/21 (57.1%)	3/16 (18.8%)
Major symptom	20/37 (54.1%)	11/21 (52.4%)	8/16 (50%)
Refractory to treatment	10/31 (32.3%)	8/21 (38.1%)	2/16 (12.5%)

### Possible risk factors for the development of pain

As IgG subclasses, titers and intra-/extrathecal distribution of autoantibodies did not seem to be associated with the development of pain, we compared the occurrence of comorbidities within groups to identify potential risk factors (see also Table 1). The prevalence of diabetes mellitus was similar in the patients without and with pain (15.4% vs. 16.2%). There was a trend for a higher prevalence of diabetes mellitus in cluster 1 (cluster 1: 23.8% vs. cluster 2: 6.2%), but statistical comparison did not reach significance. Peripheral neuropathies were more often found in patients with painful conditions, especially in those of cluster 1 (painless vs. painful: *p* = 0.01, cluster 1 vs cluster 2: *p* = 0.1). Neuromyotonia as another symptom of hyper-excitability was also more prevalent in patients with pain, but without any difference in the two clusters (painless vs. painful: *p* = 0.0003). A history of chronic back pain and/or spinal surgery was reported by 8/65 (12.3%) patients without pain and 9/37 (24.3%) patients with pain pointing to a trend of a higher prevalence in patients with pain, but the difference was not significant.

## Discussion

Assessment and characterization of painful states in patients with anti-Caspr2 autoantibodies that were included in the GENERATE database showed that pain is a relevant symptom in a large proportion of patients, is often severe and may be the major symptom. The pain phenotype differed between patients. However, two patterns could be observed: distal-symmetric burning or radiating back pain and widespread pain, often localized in the muscles associated with cramps. We did not find any association with autoantibody-related factors like titer, IgG subclass, or intrathecal autoantibody synthesis. Instead, certain potential risk factors for chronic pain like peripheral neuropathy, or preexistent chronic back pain as well as neuromyotonia tended to occur more frequently in patients with anti-Caspr2 autoantibodies and pain, arguing in favor of hyper-excitability, preexisting sensitization and/or decreased pain thresholds to play a role in pain induction.

The prevalence of pain in our study cohort is consistent with previous studies that also reported neuropathic pain in 30 to 60% of adult patients and often severe intensity of pain [2, 4, 7, 8, 14]. Painful phenotypes were not associated with different titers or certain IgG subclasses. Thus, there were no evident differences on the autoantibody level that could explain different clinical phenotypes. In contrast to recent studies, that had reported that in patients with neuropathic pain, autoantibodies are only found in the serum whereas in patients with a CNS involvement they are also positive in the CSF, we could not find any differences between patients with and without intrathecal synthesis of autoantibodies and also no association to CSF pleocytosis. This may be explained by the fact that all our patients also had CNS symptoms.

Existing data on the clinical phenotype of pain are rare and may be biased as several studies only included neuropathic pain. Hence, pain states that are not clearly neuropathic (like arthralgia or myalgia) may not have been included: A systematic study using pain questionnaires depicted variable pain qualities and locations, but no uniform phenotypes [7]. Other more descriptive studies and case series often described distal burning pain (as also reported by a relevant proportion of the patients in our cohort) but also back pain, muscle pain and in a pediatric cohort even abdominal pain [3, 8, 9, 15]. Thus, variable pain phenotypes occur in patients with anti-Caspr2 autoantibodies and from a purely clinical point of view do not even clearly correspond to neuropathic pain. From a pathophysiological point of view, regarding hyper-excitability of nociceptive neurons, i.e., peripheral sensitization to pain, as the cause of these pain conditions, pain in patients with anti-Caspr2 autoantibodies may better fit into the newer category of nociplastic pain that was introduced by the International Association for the Study of Pain to differentiate pain with altered nociception from pain that is caused by a distinct lesion to the nervous system [16]. Indeed, the existence of chronic widespread pain and hypersensitivity may argue in favor of this classification [17].

As neither presence or absence of pain nor the pain phenotypes in our study was related to autoantibody titers, IgG subclasses, or intra-/extrathecal autoantibody synthesis, we searched for patient-related factors that may explain these differences, i.e., risk factors for certain pain conditions that may be symptomatic due to peripheral sensitization by autoantibody binding. By clustering patients into two patterns of pain phenotypes, we noted that neuromyotonia appeared to be most frequent in patients with myalgia, whereas a preexisting peripheral neuropathy and diabetes mellitus appeared to be associated with distal-symmetric “small-fiber-neuropathy-like” phenotype. However, larger cohorts of these subgroups are needed to definitely establish risk factors.

In summary, our data confirm pain to be a relevant symptom in patients with anti-Caspr2 autoantibodies, which may be the major or even only symptom and suggest a broad range of pain conditions that can be categorized into two clusters but cannot be defined by IgG subclasses, titers, or CSF/serum positivity. Prospective studies on large cohorts using prespecified pain questionnaires and systematic screening for risk factors are needed to further support our findings. Testing for anti-Caspr2 should be considered in patients with unclear pain, not only in unambiguously neuropathic pain description but also in patients with widespread muscle pain, particularly in combination with muscle cramps.

### Supplementary Information

Below is the link to the electronic supplementary material.

Supplementary file1 (XLSX 19 KB)

## Data Availability

The data that support the findings of this study are available from the corresponding author upon reasonable request.
